# Development and implementation of patient-level prediction models of end-stage renal disease for type 2 diabetes patients using fast healthcare interoperability resources

**DOI:** 10.1038/s41598-022-15036-6

**Published:** 2022-07-04

**Authors:** San Wang, Jieun Han, Se Young Jung, Tae Jung Oh, Sen Yao, Sanghee Lim, Hee Hwang, Ho-Young Lee, Haeun Lee

**Affiliations:** 1 Enolink, Cambridge, USA; 2 Department of Family Medicine, Seoul National University Bundang Hospital, Seongnam, Republic of Korea; 3 Department of Digital Healthcare, Seoul National University Bundang Hospital, 172 Dolma-ro, Bundang-gu, Seongnam, 13620 Republic of Korea; 4 Department of Internal Medicine, Seoul National University Bundang Hospital, Seongnam, Republic of Korea; 5 Department of Internal Medicine, Seoul National University College of Medicine and Seoul National University Bundang Hospital, 82 Gumi-ro, Bundang-gu, Seongnam, 13620 Republic of Korea

**Keywords:** Endocrinology, Health care, Medical research

## Abstract

This study aimed to develop a model to predict the 5-year risk of developing end-stage renal disease (ESRD) in patients with type 2 diabetes mellitus (T2DM) using machine learning (ML). It also aimed to implement the developed algorithms into electronic medical records (EMR) system using Health Level Seven (HL7) Fast Healthcare Interoperability Resources (FHIR). The final dataset used for modeling included 19,159 patients. The medical data were engineered to generate various types of features that were input into the various ML classifiers. The classifier with the best performance was XGBoost, with an area under the receiver operator characteristics curve (AUROC) of 0.95 and area under the precision recall curve (AUPRC) of 0.79 using three-fold cross-validation, compared to other models such as logistic regression, random forest, and support vector machine (AUROC range, 0.929–0.943; AUPRC 0.765–0.792). Serum creatinine, serum albumin, the urine albumin-to-creatinine ratio, Charlson comorbidity index, estimated GFR, and medication days of insulin were features that were ranked high for the ESRD risk prediction. The algorithm was implemented in the EMR system using HL7 FHIR through an ML-dedicated server that preprocessed unstructured data and trained updated data.

## Introduction

Type 2 diabetes mellitus (T2DM) is known to be a leading cause of end-stage renal disease (ESRD) worldwide^1,2^. ESRD is the final and permanent stage of chronic kidney disease (CKD), where kidney function has declined to the point which the kidneys cannot function any longer on their own^3^. As the population of individuals with T2DM is increasing rapidly, the population of individuals with ESRD is also accelerating^4–6^. In Korea, the prevalence of T2DM increased over 17 years from 2001 (8.6%) to 2018 (13.8%) in adults ≥ 30 years of age^7^. This prevalence is not much different from that of adults in the United States^8^. Furthermore, although many anti-diabetic and anti-hypertensive medications have been developed, the prevalence of ESRD has not decreased^9^.

Recent studies have proven that at least 90% of patients with diabetic kidney disease (DKD) are at a higher risk of mortality because of comorbidities such as cardiovascular disease and kidney failure^10^. Furthermore, in contrast to other diabetic complications, mortality associated with renal complications is continuously increasing^11^. Therefore, there is a need to predict and prevent ESRD, which is the most severe stage of DKD, to slow or stop the progression of DKD by performing early diagnosis and treatment, thereby minimizing the medical costs associated with kidney failure treatment.

Many studies have focused on determining the predictive factors for the development of DKD, including clinical markers such as baseline values of the glomerular filtration rate (GFR), systolic blood pressure, fasting blood glucose, triglycerides^12^, and genetic markers, such as the angiotensin-converting enzyme genotype^13^. Additionally, various risk prediction models have been developed that incorporate these known risk factors^14–18^. These studies successfully presented the risk prediction for DKD. For example, one study conducted in China with 8.3 years of follow-up predicted the 3-year, 5-year, and 8-year risk of ESRD in type 2 diabetes patients with good accuracy (area under the receiver-operating characteristic [AUROC] curve of 0.90, 0.86, and 0.81, respectively) using discriminatory values such as age, sex, age at the time of diabetes onset, creatinine, albuminuria, variations in HbA1c, combined statuses of hypertension, diabetes, and hyperlipidemia in risk-scoring systems^14–16^.

However, variables other than the known risk factors can also influence the development of ESRD. Thus, machine learning using various risk factors should be adopted to improve the accuracy of the predictive model for ESRD, as in the case of previous studies where machine learning was applied to increase accuracy in diagnosing T2DM^19,20^. To our knowledge, no previous study has used machine learning to develop a patient-level ESRD risk prediction model. Furthermore, any attempt to deploy such a prediction model in the real-world clinical setting such that it is of service to patients and clinicians has been deficient. Integrating the prediction model into the electronic medical records (EMR) system would provide additional benefits such as allowing the identification of patients at a high risk of ESRD in busy hospital environments.

In this study, we aimed to develop a patient-level prediction model for ESRD in adults with type 2 diabetes mellitus that presents a risk score for developing ESRD within 5 years. We also aimed to distinguish the model from similar pre-existing tools, such as the Kidney Failure Risk Equation and the tools from the Chronic Kidney Disease Prognosis Consortium^21,22^. Furthermore, we aimed to create a model that is applicable in actual clinical practice in a multitude of hospitals and can be implemented in the EMR system.

## Results

### Clinical characteristics of the patients

The baseline characteristics of 19,159 individuals in the cohort are presented in Table 1. During 16 years of follow-up, 1,583 patients (8.3%) developed ESRD. These patients were older and had higher blood pressure and poor lipid profiles compared to individuals without ESRD. However, patients with ESRD were less obese than their counterparts.

**Table 1:** Baseline characteristics of study participants.

	Total	Not progressed to ESRD	Progressed to ESRD	P value
Number	19,159	17,576	1,583	
Age	62.3 ± 11.7 (100.0%)	62.0 ± 11.6 (100.0%)	66.1 ± 12.1 (100.0%)	< 0.001
**Sex**				
Male	10,674 (55.7%)	9739 (55.4%)	935 (59.1%)	
Female	8485 (44.3%)	7837 (44.6%)	648 (40.9%)	0.005
**Current smoking**				
No	10,052 (52.5%)	9178 (52.2%)	874 (55.2%)	
Yes	3622 (18.9%)	3281 (18.7%)	341 (21.5%)	0.192
Duration of Hospital Visits (days)^a^	1260.5 ± 1061.0 (100.0%)	1267.6 ± 1042.5 (100.0%)	1181.8 ± 1245.2 (100.0%)	0.002
Weight (kg)	66.0 ± 12.3 (88.3%)	66.2 ± 12.2 (88.2%)	63.8 ± 12.6 (88.6%)	< 0.001
BMI (kg/m^2^)	25.2 ± 4.2 (56.4%)	25.2 ± 4.1 (57.2%)	24.7 ± 5.3 (47.9%)	0.003
SBP (mmHg)	129.1 ± 17.7 (89.4%)	128.6 ± 17.2 (89.3%)	135.1 ± 21.3 (90.1%)	< 0.001
DBP (mmHg)	74.1 ± 11.4 (90.6%)	74.2 ± 11.3 (90.5%)	73.0 ± 12.6 (91.9%)	< 0.001
TC (mg/dL)	170.4 ± 37.7 (99.1%)	170.8 ± 37.1 (99.2%)	165.8 ± 44.4 (98.8%	< 0.001
HDL (mg/dL)	48.5 ± 12.2 (85.1%)	48.7 ± 12.1 (85.6%)	45.2 ± 12.6 (79.5%)	< 0.001
LDL(mg/dL)	92.2 ± 29.0 (78.0%)	92.1 ± 28.6 (78.7%)	93.0 ± 33.8 (70.9%)	0.324
TG (mg/dL)	144.7 ± 80.6 (85.1%)	144.0 ± 80.2 (85.6%)	152.7 ± 85.5 (79.6%)	< 0.001
eGFR (ml/min/1.73 m^2^)	76.7 ± 22.4 (98.9%)	79.9 ± 19.3 (98.9%)	41.5 ± 24.0 (98.9%)	< 0.001
UACR	72.7 ± 216.9 (51.9%)	45.3 ± 145.6 (52.4%)	420.6 ± 494.4 (45.6%)	< 0.001
Duration of Type 2 Diabetes^b^	884.5 ± 901.4 (100.0%)	884.3 ± 884.2 (100.0%)	886.4 ± 1074.2 (100.0%)	0.930
**Hypertension**				
No	2446 (12.8%)	2422 (13.8%)	24 (1.5%)	< 0.001
Yes	16,713 (87.2%)	15,154 (86.2%)	1559 (98.5%)	
Duration of hypertension	1001.9 ± 973.5 (87.2%)	1009.8 ± 959.1 (86.2%)	924.7 ± 1101.0 (98.5%)	0.001

Data are shown in mean ± SD or number (%).The percentage in the parenthesis indicates the percentage of non-zero values (continuous variables) or the percentage of a given category (categorical variables). Patient characteristics are calculated from one randomly selected cohort.

*ESRD* end stage renal disease, *BMI* body mass index, *SBP* systolic blood pressure, *DBP* diastolic blood pressure, *TC* total cholesterol, *HDL* high density lipoprotein, *LDL* low density lipoprotein, *TG* triglyceride, *T2D* type 2 diabetes, *UACR* Urine albumin to creatinine ratio, *eGFR* estimated glomerular filtration rate.

^a^Duration of Hospital Visits is the total duration of patients’ visits in Seoul National University Bundang Hospital.

^b^The total duration of T2DM management gained through ICD-10 diagnosis codes of Type 2 Diabetes.

### Model discrimination and calibration performance

Our model had good discriminatory power, which indicates how well our model discriminates between patients with and without ESRD, with an AUROC curve of 0.947 and area under precision recall curve (AUPRC) of 0.785 (Table 2).

**Table 2:** Model performance of the developed model.

Model	XGB				
Metric	Accuracy	AUPRC	AUROC	Precision	Recall
Count	9.000	9.000	9.000	9.000	9.000
Mean	0.959	0.785	0.947	0.828	0.631
SD	0.002	0.013	0.005	0.018	0.024
ci_low	0.957	0.777	0.944	0.817	0.616
ci_high	0.960	0.794	0.950	0.840	0.644

The values are derived from 9 iterations, which is the number of fold (k = 3) * the number of epochs (N = 3). Final performance value is the average of three-fold validation over three cohorts. The confidence interval is calculated by bootstrapping first then calculating the 95 percentile range.

*ci_low* Lowest 95% confidence interval of experiments, *ci_high* Highest 95% confidence interval of experiments, *SD* Standard Deviation, *AUPRC* Area Under Precision-Recall Curve, *AUROC* Area Under Receiver-Operator Characteristics.

**Figure 1:**
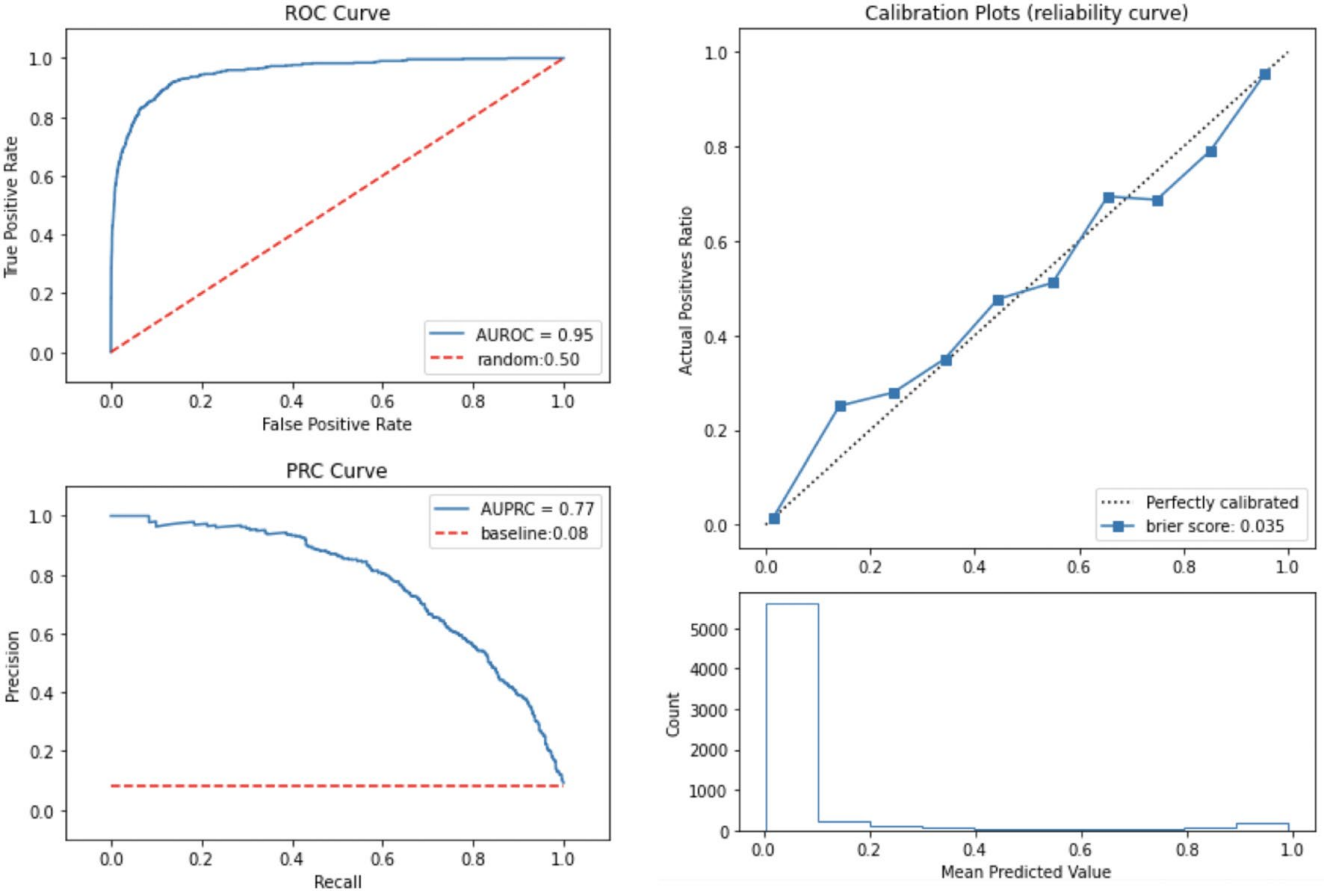
Model discrimination and calibration performance. *AUROC* area under the receiver-operating characteristic, *PRC* precision recall curve, *ROC* receiver-operating characteristic.

The calibration performance was also assessed with the calibration plot^23^. The plot was created by discretizing the [0, 1] interval into 10 uniform bins. For each bin, the mean predicted probability and true fraction of positive cases were plotted on the x-axis and y-axis, respectively. A perfectly calibrated model is represented by a diagonal line. If the model was above the diagonal line, it indicated that the model underestimated the risks. Similarly, if the model was below the diagonal line, it was an indication of an overestimation of risks. We observed that our model moderately overestimated higher risk score groups (Fig. 1).

### Model comparison

We compared the discriminatory power of the XGBoost model against other types of models, such as linear regression, support vector machine, decision tree, and random forest models (Table 3 and Supplementary Fig. 1). Our XGBoost model had the best discrimination power (AUROC curve, 0.947; AUPRC, 0.792) compared to other models (AUROC curves range, 0.929–0.943; AUPRC range, 0.765–0.792).

**Table 3:** Model performances of difference machine learning algorithms.

Model	LR		RF		SVM		XGB	
	AUPRC	AUROC	AUPRC	AUROC	AUPRC	AUROC	AUPRC	AUROC
count	9.000	9.000	9.000	9.000	9.000	9.000	9.000	9.000
mean	0.783	0.942	0.792	0.943	0.765	0.929	0.792	0.947
SD	0.012	0.002	0.015	0.005	0.011	0.007	0.009	0.004
ci_low	0.775	0.940	0.782	0.939	0.759	0.924	0.786	0.945
ci_high	0.790	0.943	0.800	0.946	0.772	0.933	0.797	0.950

*ci_low* Lowest 95% confidence interval of experiments, *ci_high* Highest 95% confidence interval of experiments, *SD* Standard Deviation, *AUPRC* Area Under Precision-Recall Curve, *AUROC* Area Under Receiver-Operator Characteristics.

### Cost–benefit analysis (decision curve analysis)

**Figure 2:**
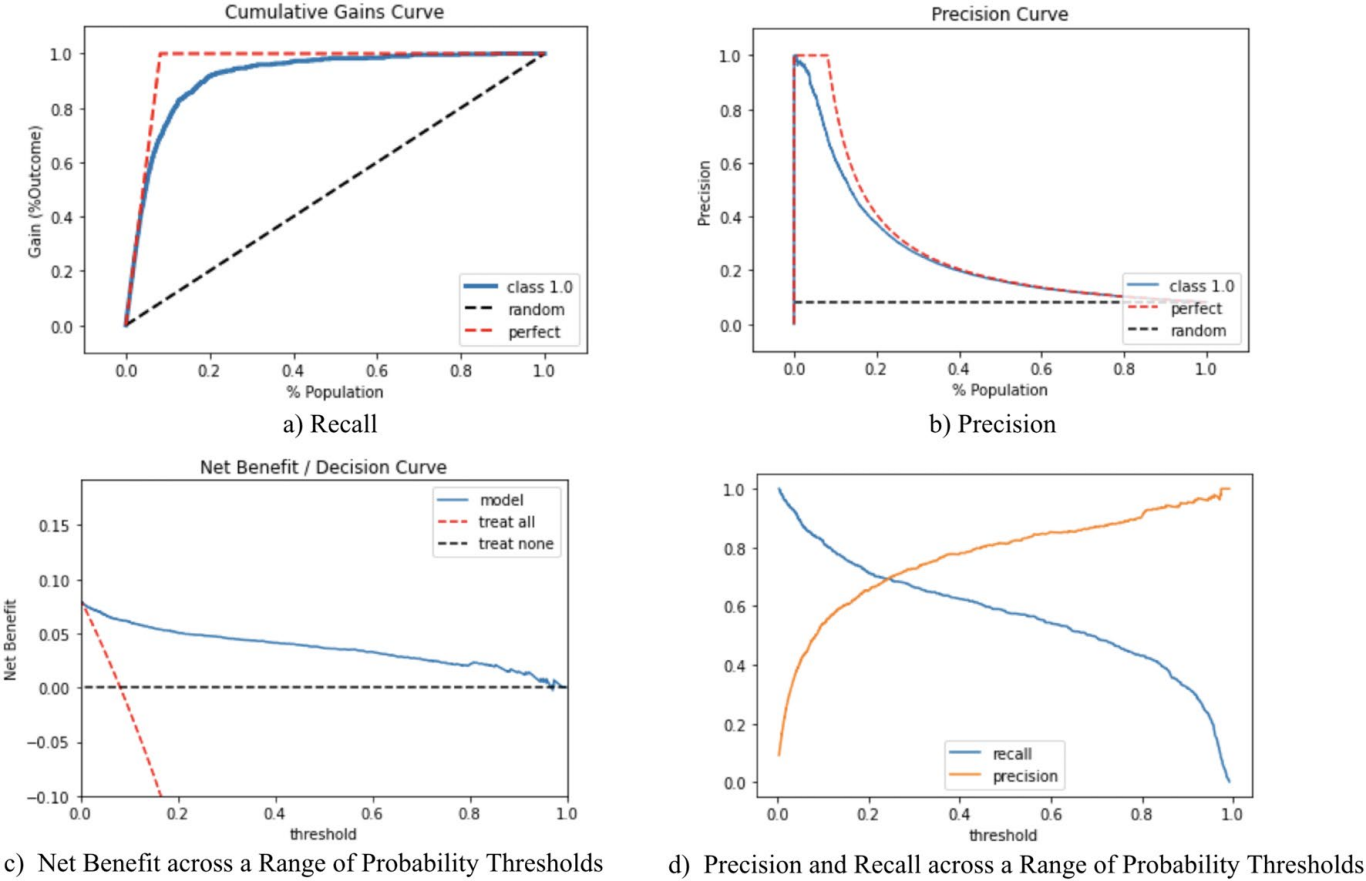
Cost–benefit analysis.

To assess the recall of our model, which indicates the proportion of true-positive cases compared to all positive cases (true-positive and false-negative cases), we plotted the recall curve (Fig. 2a), which shows the recall rate as a function of the number of cases treated.

Similarly, we also assessed the precision of the model, which is the proportion of true-positive cases compared to all predicted cases, by ranking predicted cases in descending order according to their predicted probabilities (Fig. 2b).

To evaluate our model performance from the perspective of clinical value, we conducted a decision curve analysis (Fig. 2c). The main advantage of the decision curve analysis is that it incorporates clinical consequences into the model evaluation and does not require additional data, such as an explicit assessment of health outcomes or treatment-related costs. Instead, it considers a threshold probability as an informative indicator of relative harms of a false-positive and a false-negative prediction^24,25^. We also analyzed changes of precision and recall across a range of probability thresholds (Fig. 2d).

### Model explainability

**Figure 3:**
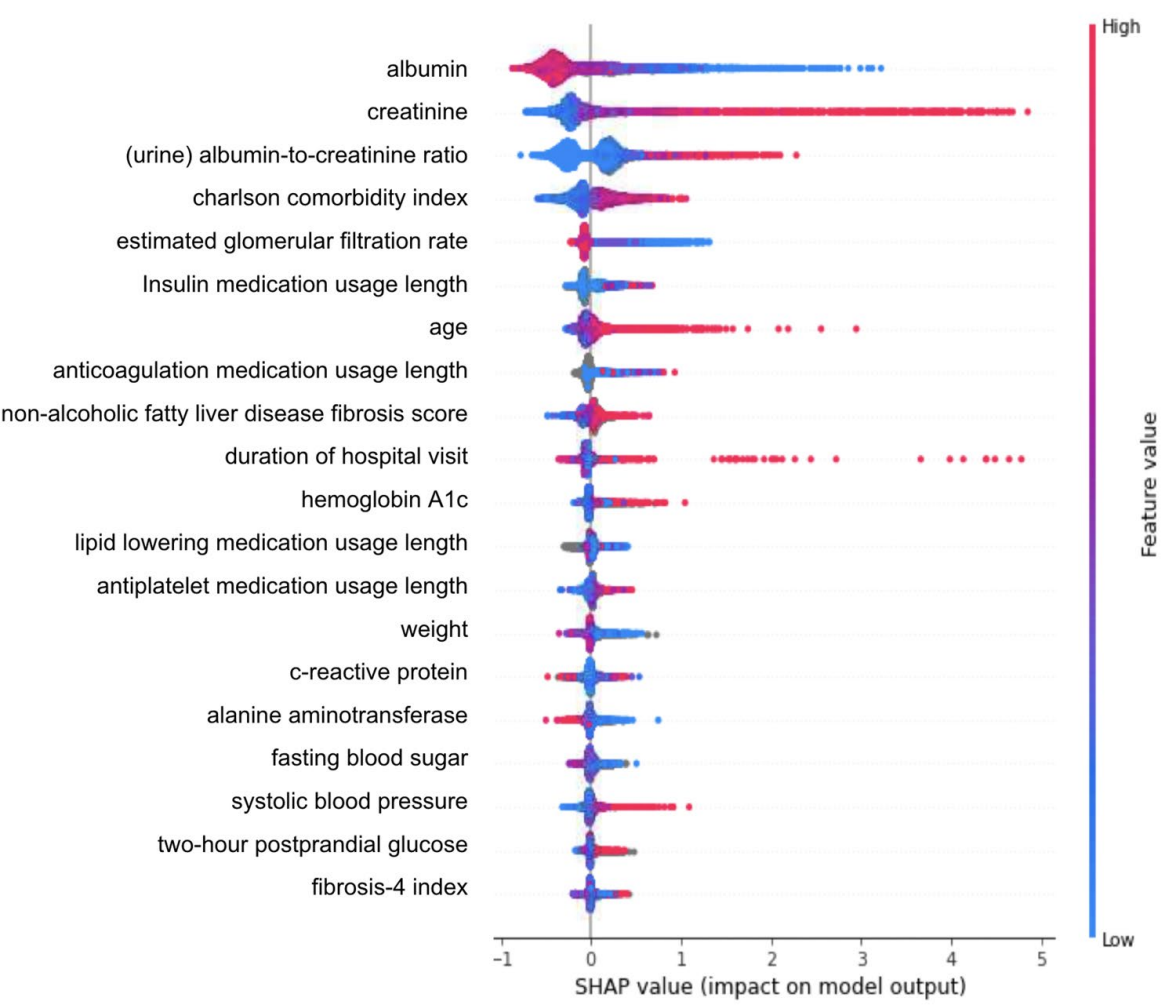
SHAP summary plot. If a feature is located on the upper side of this figure, then it implies a higher contribution of the feature to the prediction. More specifically, each dot represents the data of each patient, and the color of the dot indicates whether the respective feature value is low or high (as shown on the y-axis on the right). The location of the dot indicates whether the feature increases (right) or decreases (left) the risk prediction. The farther a dot is from 0, the greater the contribution to the prediction. *SHAP* Shapley Additive Explanations.

We used the Shapley Additive Explanations (SHAP) analysis to interpret the data acquired from the machine learning process of the XGBoost model and evaluate the importance of the individual features of the prediction of ESRD. SHAP is a framework that interprets predictions that a machine learning model conducted. It calculates the contribution of each feature to the prediction and assigns an importance value to each feature depending on the result of the calculation^26^. Figure 3 shows the magnitude and direction of the contribution of each feature compared to the average model prediction.

To assess the stability of the importance of these features, we checked the ranges and standard deviations of their rankings with regard to different index dates (Supplementary Fig. 2). According to the results of iterative experiments, the features with a high rank with the greatest importance did not show much distinction from those with other ranks. Serum creatinine, serum albumin, the urine albumin-to-creatinine ratio, Charlson comorbidity index, estimated GFR, and medication days of insulin were features that were ranked high for the ESRD risk prediction^27^. However, our prediction model incorporated new parameters such as blood albumin level, and non-invasive markers of hepatic fibrosis such as nonalcoholic fatty liver disease, fibrosis score, and fibrosis-4 index.

### Implementation of the machine learning-based clinical decision support system

The developed model was implemented with a dedicated server for the Machine Learning-based Clinical Decision Support System (Supplementary Fig. 3). The server extracted structured data through the Fast Healthcare Interoperability Resources server from EMR, such as visit history, medication history, laboratory values, and vital signs (Supplementary Table 2). For the extraction of unstructured data, such as smoking history, reading of imaging studies, and results of electrocardiography, the server directly accessed the EMR and preprocessed the unstructured data into structured input features for the calculation of the prediction model based on the XGBoost algorithm.

### Dashboard

A prototype of a comprehensive dashboard for the Machine Learning-based Clinical Decision Support System was designed to provide the results of the prediction algorithm and related test results of an individual patient. The ESRD risk determined by the prediction model was presented with the SHAP analysis to enable users to identify modifiable risk factors among the high-ranking input features according to the SHAP results (Supplementary Fig. 4).

## Discussion

We successfully developed a 5-year ESRD risk prediction model for type 2 diabetes mellitus using a machine learning algorithm based on the medical data of the study cohort consisting of 19,159 patients. Among various machine learning methods, the XGBoost classifier showed the best discriminatory performance when processing medical data. Additionally, we applied the SHAP analysis to evaluate the relative importance of each feature, which could provide specific medical information to physicians.

Current treatment guidelines for chronic kidney disease in patients with diabetes have suggested stratifying the patient’s risk according to GFR and albuminuria categories^28^. However, this classification is too simple to correctly predict the individual risk of ESRD. Precise prediction of the prognosis of renal function is necessary when deciding whether to refer the patient to a nephrologist, preparing a long-term plan (e.g., renal transplantation), and providing appropriate medical intervention. Our model provided the 5-year risk of ESRD with good discrimination power. Additionally, the risk factors for ESRD that we identified in our model are well-known, which means our model is clinically explainable. A decreased serum albumin level was one of the strongest predictive factors in our model. The albumin level might represent the patient’s nutritional status and could be a marker for the poor prognosis of chronic kidney disease^29^. Systemic inflammation in a critically ill patient is known to cause altered albumin homeostasis and lead to hypoalbuminemia^30^. Additionally, hepatic fibrosis indices were also introduced in our model, which could be related to advanced stages of diabetes complications^31,32^. Nonalcoholic fatty liver disease has been proven to accelerate the decline in kidney function in chronic kidney disease patients through the activation of a pathway that enhances the transcription of pro-inflammatory genes and amplification of immunologic inflammatory responses^33^.

The high AUROC curve showed that our model was sufficiently able to distinguish between positive and negative cases. The high AUPRC of our model also provided significant benefits because it was much higher than the prevalence of ESRD in the training data. The recall rate of our model with a default threshold of 0.5 (i.e., treating patients when the risk score is higher than 0.5) had a 95% confidence interval between 0.62 and 0.64. If a physician provides treatment for patients with a risk score of more than 0.5, then this plan would treat 62–64% of patients who will develop ESRD. Physicians can always adjust the threshold based on their case and depending on the desired levels of recall and precision (Fig. 2d). For example, if physicians would like to increase the coverage (recall) to 80%, they may use a risk score cutoff of 0.1 while still maintaining a precision value as high as 0.6.

Similarly, the precision of our model had a 95% confidence interval between 0.82 and 0.84. Therefore, if the model predicts a patient with T2DM will have ESRD, 82–84% of them will actually have ESRD in the future.

Compared to a conventional model from cox regression by Tangri et al.^34^, our model has several advantages. First, our model predicts ESRD, not CKD stages 3 to 5 as in the conventional model, which means our model targets most severe case of CKD in T2DM patients. Second, new predictors such as serum albumin and makers of fatty livers, which were ranked high in the SHAP analysis. Third, our model can be used directly with EHR systems using FHIR resources.

Although our model provided significant improvements in terms of discrimination and calibration performance for ESRD risk prediction, there are several areas that can be improved. First, several features related to disease or medication were engineered as binary indicators. However, their effects can vary depending on the severity of other comorbidities or the dosage of medication, which were not fully captured in our model. Second, although the classification modeling approach using XGBoost enabled us to handle missing values, the drawback was that the model required every patient to have sufficient observation periods (i.e., 5 years) to correctly identify outcome labels. Therefore, the most recent index date that could be used in our model was at least 5 years before the last date of the available data; consequently, almost 10 years of EMR data could not be used in our model. Third, no imputations were performed to handle missing data, which could have impacted the quality of the modeling process. Future work should consider incorporating data imputation. Fourth, we did not reflect the definition of a period in which of eGFR < 15 ml/min/1.73 m^2^ and the need for dialysis persist for more than 3 months to accurately define ERSD. It might overestimate the incidence of patients with ESRD in the study cohort. Finally, we did not validate our model for the independent data set and prove the clinical effectiveness of our model Therefore, we cannot generalize our results to other environments. Further study is needed to evaluate accuracy and clinical effectiveness of the developed model in other environments.

Despite these limitations, our model provides several meaningful clinical and practical implications. The inclusion of additional data items from the EMR system contributed to its better performance. Previous studies could not find significant improvements in model performance by incorporating more variables beyond the traditional chronic kidney disease risk model variables^14^. It would be beneficial for future studies to assess the extent to which additional EMR data items contributed to the improvement in performance. Our model will be a great resource for physicians to assess the developmental and progression risk of ESRD within the next 5 years for type 2 diabetes mellitus patients, and it will help them make appropriate treatment decisions to prevent or slow the progression of ESRD.

## Methods

### Development of models

The development of the ESRD prediction model was accomplished through six steps. First, the study cohort was selected from the initial dataset by applying the inclusion and exclusion criteria. Second, we assigned a random index date for every patient in the cohort. Third, to identify patients with ESRD, a primary composite outcome of the study, we labeled the outcome and prediction timeline according to the clinical criteria described. Fourth, as a result of processing the time-series data of patients using the eight feature generators, 49 features were retained to compose the ESRD prediction model. Subsequently, we trained and validated the model through k-fold cross-validation. Finally, we repeated step 1 through step 4 N times; therefore, the performance of the prediction model could be based on the average of K*N iterations.

To select the study cohort, we used the medical data extracted from the EMR system of Seoul National University Bundang Hospital, a tertiary academic hospital in South Korea. Seoul National University Bundang Hospital developed and adopted an electronic health system in 2003. Of 61,936 patients from the initial dataset, 60,909 patients remained after excluding those without at least three outpatient visit records. Thereafter, we continued constructing the final dataset by applying our inclusion criteria and exclusion criterion for the ESRD prediction model.

**Figure 4:**
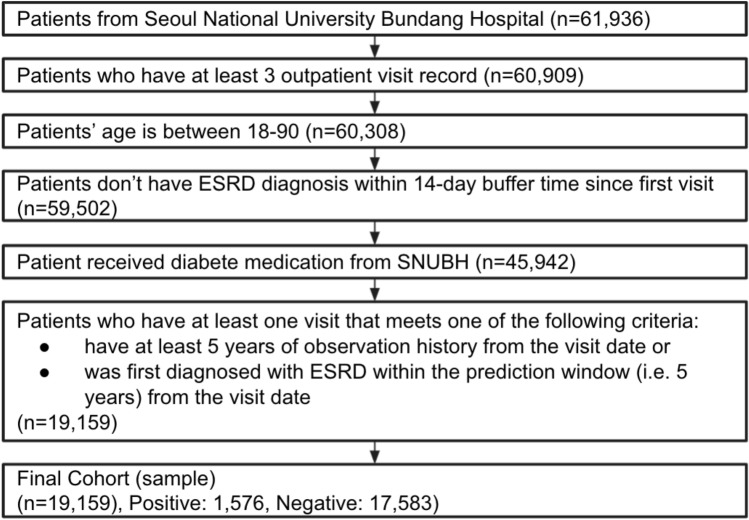
Cohort selection. *ESRD* end-stage renal disease, *SNUBH* Seoul National University Bundang Hospital.

Our inclusion criteria were as follows: age between 18 and 90 years on the index date, no history of ESRD or dialysis before the index date, more than one type 2 diabetes mellitus medication record before the index date, and a history of more than 5 years of observation after the index date or first diagnosis of ESRD within the prediction window after the index date. Our exclusion criterion was a diagnosis of ESRD within 14 days of the first visit. As a result of including only patients who satisfied these criteria, we obtained a final cohort of 19,159 patients (Fig. 4).

The primary composite outcome of the study was ESRD. To identify patients with this comorbidity, we precisely defined ESRD as a diagnosis including the ICD-10 diagnosis codes N18.5 and N18.6, a history of dialysis treatment, a history of renal transplantation with ICD-9 operation codes 55.6 and 55.69, a history of continuous ambulatory peritoneal dialysis catheter insertion with ICD-9 operation codes 38.95 and 39.43, and an estimated GFR < 15 mL/min/1.73 m^2^^35,36^.

**Figure 5:**
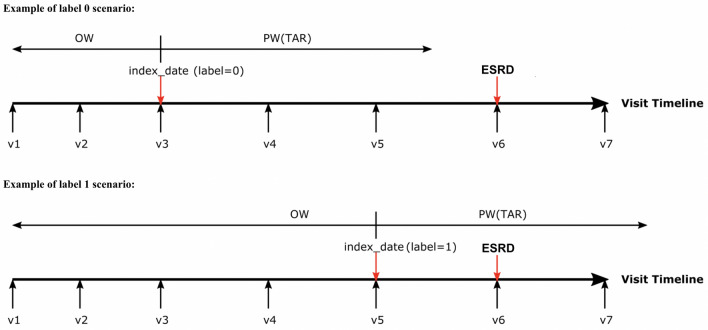
Outcome labeling examples. *OW* observation window, *PW* prediction window, *TAR* time at risk.

Next, for each patient, we randomly selected one index date to construct a cohort. For each patient in the cohort with a selected index date, we labeled the outcome as 1 if the patient developed the comorbidity of interest during the prediction window (5 years); in other cases, the outcome was labeled as 0. Consider the scenarios depicted in Fig. 5. The horizontal line represents the timeline of a patient’s hospital visits, which are denoted as v1, v2, and so on. Both scenarios in Fig. 5 represent the same patient, who was diagnosed with the comorbidity during v6. Depending on the selection of the index date for this patient, the outcome can be labeled as 0 or 1. If v3 was considered as the index date, then it was considered that this patient will not develop the comorbidity because the comorbidity was not observed within the prediction window for the given time at risk. However, if we considered v5 as the index date, then it was considered that this patient will develop the comorbidity because the prediction window includes the visit when the comorbidity was diagnosed.

We used the eight feature generators to process the time-series data of 19,159 patients. Although 52 features were initially generated, three (cacs_ewma, imt_max, and lipoprotein_ewma) were excluded because their values for more than 90% of patients were missing. Therefore, a total of 49 features were obtained for our ESRD prediction model. For handling time depending variables, we used most recent value, exponential weighted moving average value, max value, or length of records depending on the characteristics of the variable. Supplementary Table 1 shows the types of feature generators we used and the actual examples of predictors, as well as how to handle multiple measures.

New predictors were included for model development. Albumin was included because lower pre-ESRD serum albumin was associated with the incidence of ESRD in previous studies^27,37^. Markers of fatty livers disease were included because non-alcoholic fatty liver disease was related to the increased risk of chronic kidney disease in previous studies^38,39^.

For model training, we split the data into training and validation sets. We trained the binary XGBoost classification models using the training set. Then, we calculated the model performance using the validation set by repeating this procedure k times (i.e., k-fold cross-validation).

The model was evaluated using N-epoch K-fold cross-validation, which first used regular K-fold validation (step 4) and then repeated step 1 to step 4 N times. The final performance was the average of these K*N iterations.

### Ethics

This research was approved by the Institutional Review Board of Human Research of Seoul National University Bundang Hospital. Informed consent was waived because of the retrospective nature of the research and the analysis used deidentified clinical data (B-1904-535-001). The present research was conducted in accordance with the Declaration of Helsinki.

## Supplementary Information

Supplementary Information.

## Data Availability

Data is not available to public due to the regulation of IRB in SNUBH. S.Y.J. should be contacted if someone wants to ask a question about the data from this study.
