# Supplementary material for: Development and implementation of patient-level prediction models of end-stage renal disease for type 2 diabetes patients using fast healthcare interoperability resources
Source: Sci Rep. 2022 Jul 4;12:11232. doi: 10.1038/s41598-022-15036-6 (PMC9253099; doi:10.1038/s41598-022-15036-6)
Supplement: Supplementary file 1 — Supplementary Information. [file 41598_2022_15036_MOESM1_ESM.docx]

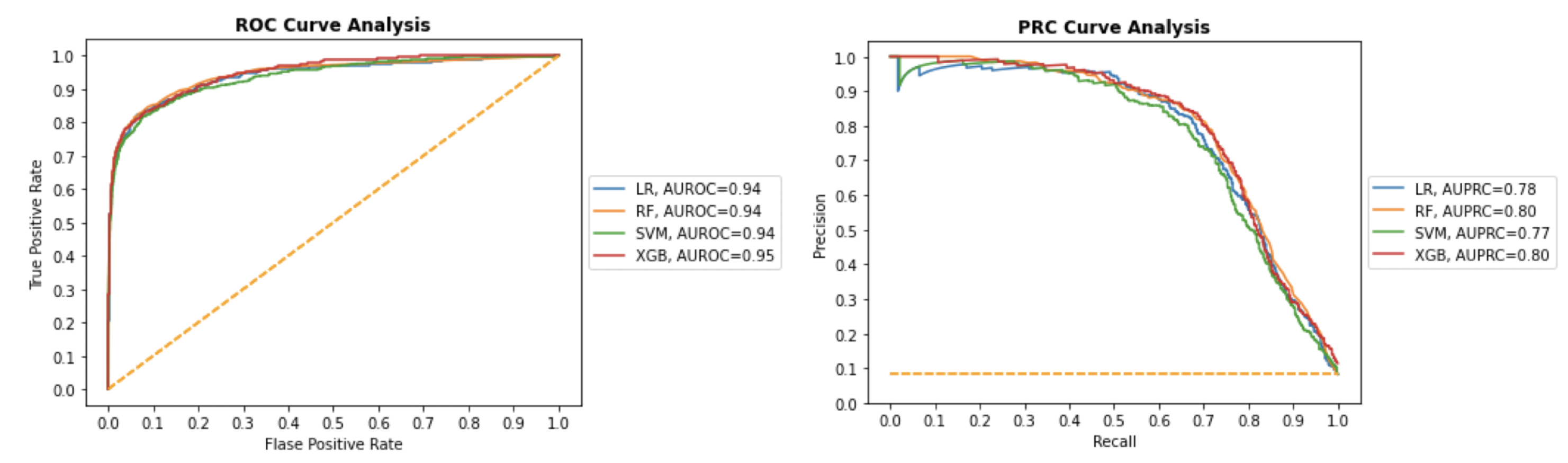


**Supplementary Figure 1.** Area Under Receiver-Operator Characteristics and Area Under Precision-Recall Curve of difference machine learning models


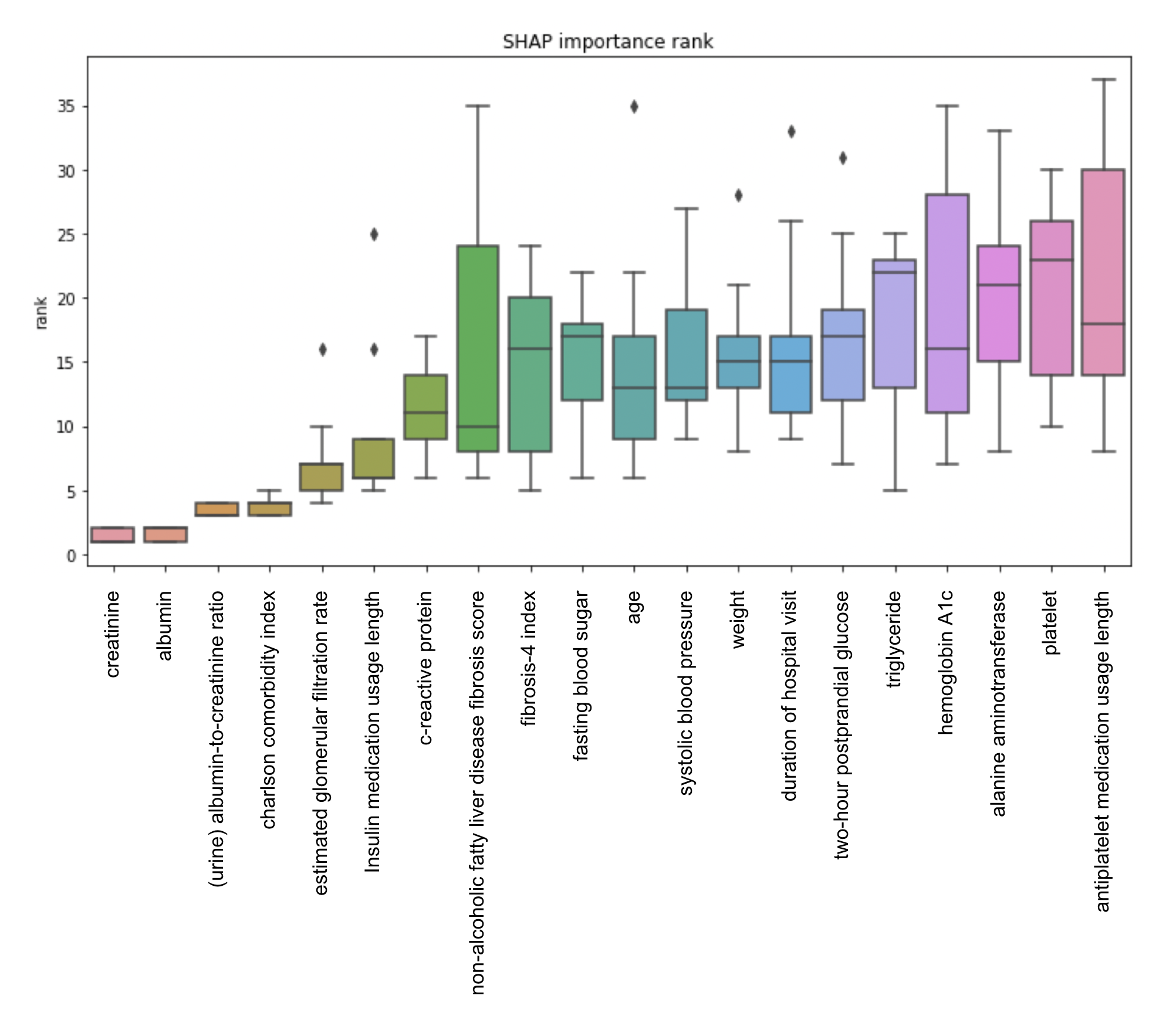


**Supplementary Figure 2.** SHAP importance rank

 
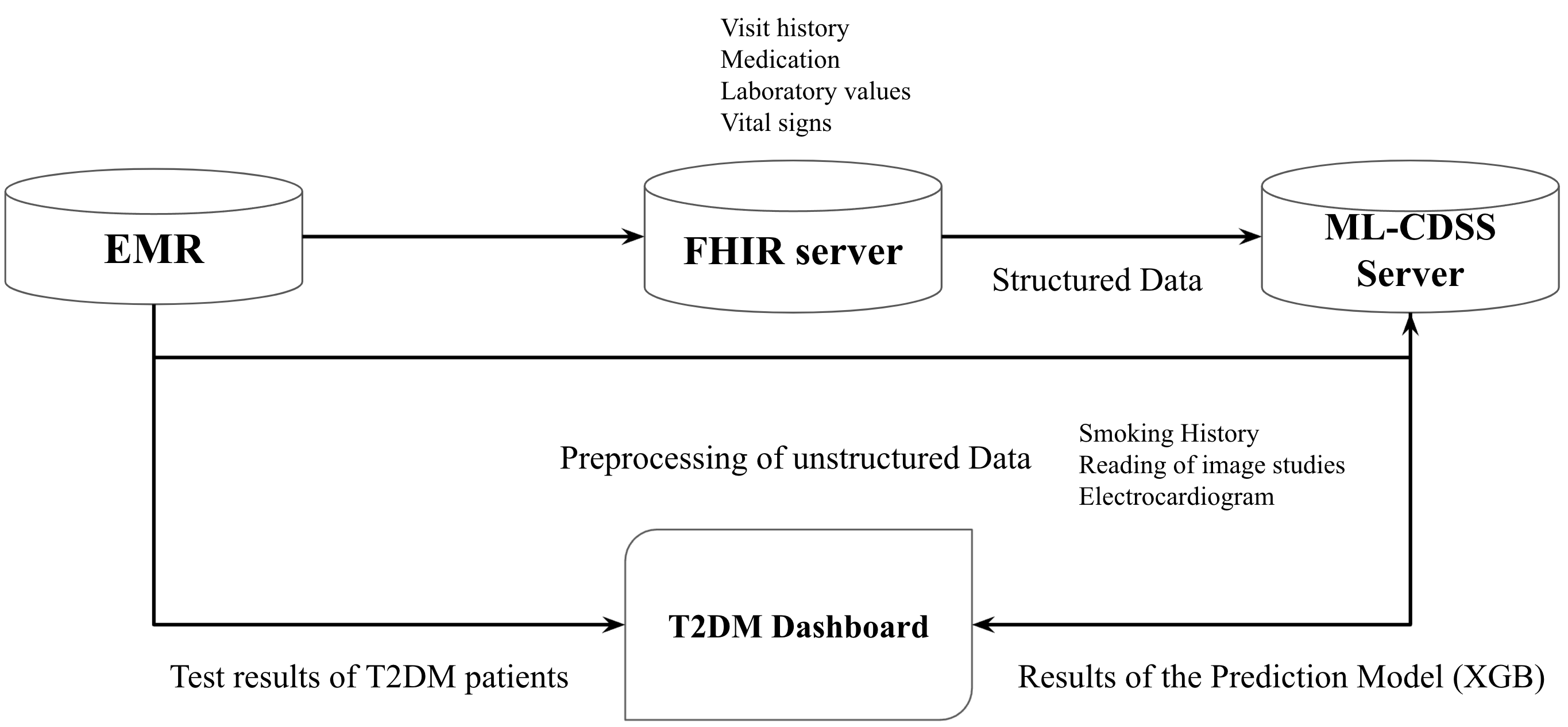


**Supplementary Figure 3.** System architecture of ML-CDSS with HL7 FHIR


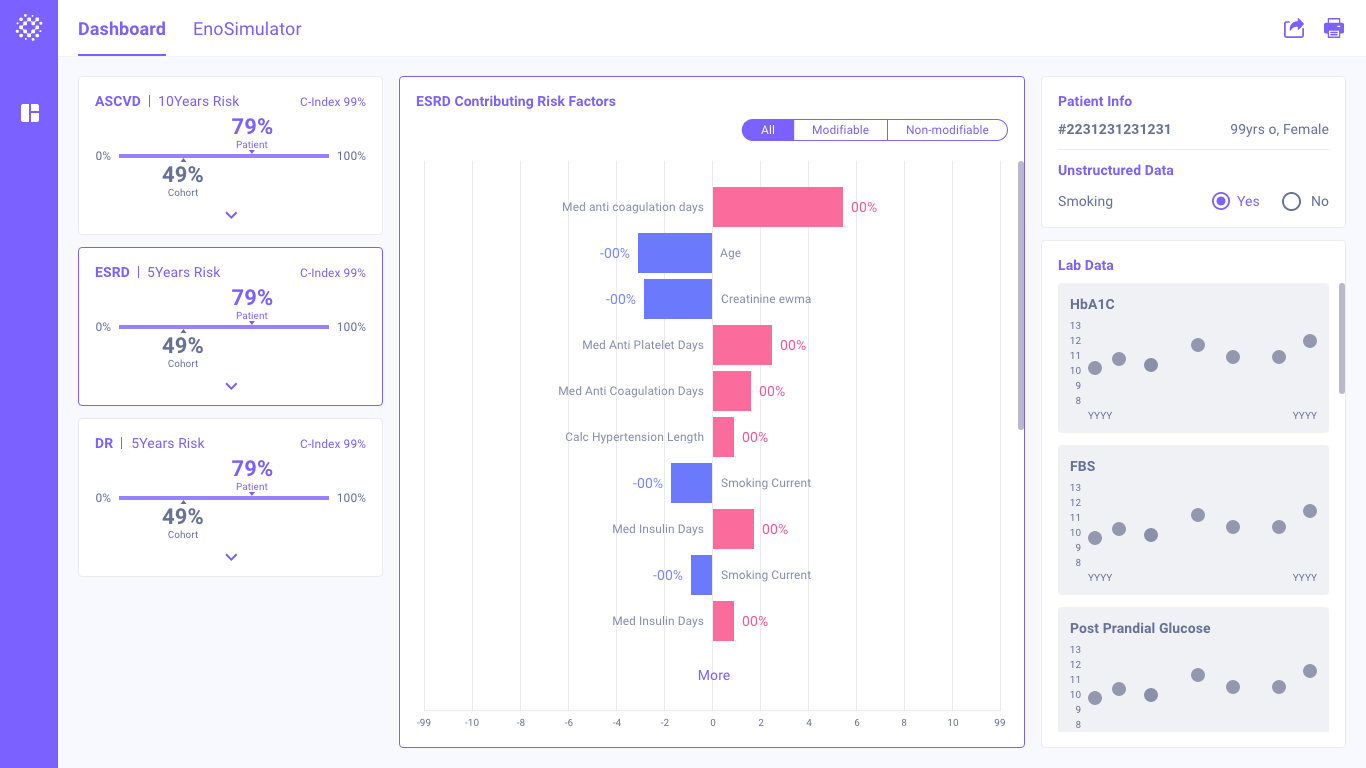


**Supplementary Figure 4.** Prototype of ML-CDSS dashboard

| **Supplementary Table 1.** Feature Generator Description | | |
| --- | --- | --- |
| Feature Generators | Description | Predictors |
| Age | Age at index date | ● Age |
| Gender | Gender of a patient | ● Gender |
| Most Recent (MR) | Calculates the most recent value before index_date | ● CCI |
| Exponential weighted moving average (EWMA) | Calculates the exponentially weighted moving average of the values before index_date (hyper-parameter alpha=0.8). | ● All measurements (23 in total)^*^  ● All liver disease surrogate markers (6 in total)  ● CACS |
| MaxValue (MV) | Calculates maximum value in time series before index_date | ● All disease progression markers except for CACS (6 in total) |
| RecordLength (RL) | Calculates number of days between first record date and index_date | ● Hypertension  ● T2DM  ● Smoking  ● Visit history |
| Record Indicator (RI) | Indicates if a record occurred before the index_date | ● Smoking (3 one-hot encoding) |
| Medication Exposure Days Total Prescription (ME) | Calculates exposure as days of all prescriptions | ● Insulin  ● Metformin  ● Anti-hypertensive  ● Lipid lowering  ● Anti-platelet  ● Anticoagulation |

^*^ 23 measurements included eight, weight, SBP, DBP, total cholesterol, HDL-cholesterol, LDL-cholesterol, triglyceride, creatinine, albumin, albumin-to-creatinine ratio, AST, ALT, insulin, hba1c, fbs, pp2, apo_a1, apo_b, lipoprotein, C-reactive protein, c-peptide, and platelets

**Supplementary Table 2**. Fast Healthcare Interoperability Resources server from EMR

| Encounter | Encounter.id  Encounter.identifier  Encounter.class  Encounter.status  Encounter.patient.reference  Encounter.patient.display  Encounter.individual.reference  Encounter.individual.display  Practitioner.identifier  Practitioner.name  Practitioner.practitionerRole.specialty.coding.code  Practitioner.practitionerRole.specialty.coding.display  Encounter.period.start  Encounter.period.end  Encounter.reason.coding.code  Encounter.reason.coding.display  Encounter.reason.coding.system |
| --- | --- |
| Medication Order | MedicationOrder.id  MedicationOrder.identifier.value  MedicationOrder.dateWritten  MedicationOrder.status  MedicationOrder.patient.reference  MedicationOrder.patient.display  MedicationOrder.encounter.reference  MedicationOrder.note  MedicationOrder.medicationReference.reference  MedicationOrder.medicationReference.display  MedicationOrder.dosageInstruction.text  MedicationOrder.dosageInstruction.timing.repeat.duration  MedicationOrder.dosageInstruction.timing.repeat.frequency  MedicationOrder.dosageInstruction.timing.repeat.period  MedicationOrder.dosageInstruction.timing.repeat.periodUnits  MedicationOrder.dosageInstruction.route.coding.code  MedicationOrder.dosageInstruction.route.coding.display  MedicationOrder.dosageInstruction.route.coding.system  MedicationOrder.dosageInstruction.doseQuantity.value  MedicationOrder.dosageInstruction.doseQuantity.unit  MedicationOrder.dosageInstruction.doseQuantity.code  MedicationOrder.dosageInstruction.doseQuantity.system |
| Observation (laboratory values, image studies, functions tests, pathology reports, Vital signs) | Observation.id  Observation.status  Observation.category.coding.system  Observation.category.coding.code  Observation.category.coding.display  Observation.code.coding.code  Observation.code.coding.display  Observation.code.coding.system  Observation.subject.reference  Observation.encounter.reference  Observation.performer.reference  Observation.performer.display  Observation.effectiveDateTime  Observation.valueString |
| Patient information | Patient.id  Patient.identifier  Patient.name.text  Patient.telecom.coding.code  Patient.gender.code  Patient.birthDate |

FHIR, Fast Healthcare Interoperability Resources

Supplementary Figure 1. Area under the receiver-operator characteristics curve (AUROC) and area under precision-recall curve (AUPRC) of different machine learning models. LR, linear regression; RF, random forest; SVM, support vector machine; XGB, XgBoost.

Supplementary Figure 2. SHAP importance rank. The smaller rank value indicates the greater importance of the feature with regard to the prediction. Min, Max, and standard deviation indicate the lowest, highest, and standard deviation of the ranks, respectively. SHAP, Shapley Additive Explanations.

Supplementary Figure 3. System architecture of the Machine Learning-based Clinical Decision Support System (ML-CDSS) with Health Level Seven (HL7) Fast Healthcare Interoperability Resources (FHIR). EMR, electronic medical records; T2DM, type 2 diabetes mellitus; XGB, XgBoost.

Supplementary Figure 4. Prototype of the Machine Learning-based Clinical Decision Support System (ML-CDSS) dashboard.
